# Unintentional drowning: Role of medicinal drugs and alcohol

**DOI:** 10.1186/s12889-017-4306-8

**Published:** 2017-05-19

**Authors:** Tuulia Pajunen, Erkki Vuori, Frank F. Vincenzi, Pirjo Lillsunde, Gordon Smith, Philippe Lunetta

**Affiliations:** 1 Department of Biomedicine, Pathology and Forensic Medicine, University of Turku, Turku, Finland; 2 Department of Forensic Medicine, University of Helsinki, Helsinki, Finland; 3 Department of Pharmacology, University of Washington, Seattle, Washington USA; 4 National Institute for Health and Welfare, Helsinki, Finland; 5 Department of Epidemiology and Public Health, University of Maryland, Maryland, USA

**Keywords:** Unintentional drowning, Autopsy, Alcohol, Psychotropic drugs

## Abstract

**Background:**

Alcohol is a well-known risk factor in unintentional drownings. Whereas psychotropic drugs, like alcohol, may cause psychomotor impairment and affect cognition, no detailed studies have focused on their association with drowning. Finland provides extensive post-mortem toxicological data for studies on drowning because of its high medico-legal autopsy rates.

**Methods:**

Drowning cases, 2000 through 2009, for which post-mortem toxicological analysis was performed, came from the database of the Toxicological Laboratory, Department of Forensic Medicine, University of Helsinki, using the ICD-10 nature-of-injury code T75.1. The data were narrowed to unintentional drowning, using the ICD-10 external-injury codes V90, V92, and W65–74. Each drowning case had its blood alcohol concentration (BAC) and concentrations of other drugs recorded. Evaluation of the contribution of psychotropic drugs to drowning was based on their blood concentration by means of a 6-grade scale.

**Results:**

Among victims ≥15 years old, unintentional drownings numbered 1697, of which, 303 (17.9%) were boating-related and 1394 (82.1%) non-boating-related. Among these, 65.0% of boating-related and 61.8% of non-boating-related victims were alcohol-positive (=BAC ≥ 50 mg/dL). The male-to-female ratio in alcohol-positive drownings was 7.3. At least one psychotropic drug appeared in 453 (26.7%) drowning cases, with some victims’ bodies showing up to 7 different drugs. Overall 70 different psychotropic drugs were detectable, with 134 (7.9%) cases both alcohol-negative and psychotropic-drug-positive, of these, 59 (3.5%) were graded 4 to 6, indicating a possible to very probable contribution to drowning. Our findings suggest that psychotropic drugs may play a significant role in drowning, in up to 14.5% of cases, independently or in association with alcohol.

**Conclusions:**

Psychotropic drugs alone or in association with alcohol may be an overlooked risk factor in drowning, due to their effects on psychomotor function and cognition. Future studies should also address other mechanisms—for instance drug-induced long-QT syndrome—by which drugs may contribute to drowning.

**Electronic supplementary material:**

The online version of this article (doi:10.1186/s12889-017-4306-8) contains supplementary material, which is available to authorized users.

## Background

Drowning is one of the world’s leading causes of unintentional deaths [1]. Many studies in high-income countries have documented the association between drowning and alcohol, especially among adult males [2–4]. By affecting the central nervous system and cognitive processes, alcohol may, for instance, cause a person to fall into water or to operate a boat in high-risk situations. In addition, it can also hamper the ability to swim and reduce survival in water [2]. A nationwide study conducted in Sweden revealed that alcohol was involved in 44% of unintentional drowning deaths [5]. Similar percentages, ranging from approximately 25 to 50, have been reported in other western countries [2, 6–8]. The estimate for Finland is as many as 60% of fatal drowning victims as being alcohol-positive, [4, 9] and as many as 54.8% have a blood alcohol concentration (BAC) over 100 mg/dL [4].

On the other hand, only limited information is available as to the role of drugs other than alcohol in unintentional drowning. The effects of many psychotropic drugs on cognition and judgment are, however, similar to those of alcohol [10]. Their impact on coordination, vision, balance, and other psychomotor functions is well documented, [11, 12] especially for vehicle driving [13, 14]. In Sweden, 22% of fatalities among unintentional drowning victims died with one or more psychotropic drugs in their blood, benzodiazepines and antidepressants being the leading drugs found, but with no indication of their role in the death [5]. Case studies have likewise reported individual drowning incidents involving particular drugs, [15] and surveys on fatal accidental injuries have also included general data on drowning and drugs [16]. However, in-depth studies addressing actual drug concentrations and aiming to assess the role of psychoactive drugs as a risk factor for drowning are lacking.

Finland (population 5.2 million) has one of the highest mortality rates by drowning among all high-income countries [4, 17, 18]. The exceedingly high rate of medico-legal autopsies, covering almost 100% of all bodies found in water, [19] with nearly 90% of the cases undergoing thorough toxicological investigations, provides an excellent opportunity to fully investigate the role in drowning deaths of psychotropic drugs [20].

This nationwide study is based on a dataset of 2828 consecutive fatal drownings occurring in Finland during a 10-year period (2000–2009) for which post-mortem (PM) toxicology was performed; unlike a similar study in Sweden, [5] independent estimates of the impairment attributable to specific drugs and their levels were available. The main aim of this survey is to evaluate the potential contributing role of psychotropic drugs, alone or in association with alcohol, in the events leading to fatal unintentional drowning.

## Methods

### Design and setting

We conducted a descriptive and retrospective study, based on individual-level data, for all drownings and their association with drugs and alcohol among Finnish residents of all ages who had undergone a medico-legal autopsy and PM toxicology, from 2000 through 2009, the last year for which detailed attribution of impairment by drug level was available.

### Data collection and analysis

Toxicological data concerning all fatal drowning cases (ICD-10 “nature of injury” code: T75.1) came from the database of the Laboratory of Forensic Toxicology, Department of Forensic Medicine, University of Helsinki. During the study period, this laboratory carried out—by statute and on request of the medical examiner performing the medico-legal autopsy-PM alcohol analyses and all other toxicology tests for the entire country. In most cases, whenever available, peripheral blood samples were from the femoral vein, and if not available, from other sites. Routine toxicological samples obtained at autopsy also include urine and vitreous humor. Blood ethanol concentration was determined by a dual-column head-space gas-chromatographic method. The final result is the average of the four independent measurements. Depending on the category of the substance in question, common and abused drugs were screened from a urine or blood sample by a relevant method. The quantitative results used in this study were always based on a blood sample. All relevant analytical methods in the laboratory (Testing Laboratory No. T115) have been accredited since 1997 by the Centre for Metrology and Accreditation according to SFS-EN 45001 ISO/IEC Guide 25. Drugs were classified according to the World Health Organization (WHO) Anatomical Therapeutic Chemical (ATC) codes [21, 22]. The study included the following variables: gender, age, manner of death (accident, suicide, homicide, undetermined intent), BAC, and blood concentrations of all drugs (up to 10) detectable in each victim.

The original data set included 2828 fatal drownings. Excluded from the data were suicidal drowning (ICD-10: X71, *n* = 546), homicide by drowning (X92, *n* = 10), and drowning of undetermined intent (Y21, *n* = 208), as well as accidental drownings with an external cause-of-injury code other than drowning (*n* = 318), e.g. land-traffic accidents and exposure to force of nature leading to drowning [18].

The present study therefore focused on 1746 accidental drownings with ICD-10 “external cause of injury” codes V90, V92, and W65–74. The 49 cases of individuals aged less than 15 years were excluded from the study. As to toxicological data, alcohol and central nervous system (CNS) drugs (ATC: N01-N06) were considered, with the exclusion of caffeine (N06BC01) and nicotine (N07BA01). We explicitly acknowledge that drugs often have effects on organ systems other than those implied by the ATC classification. Drowning deaths with BAC values ≥50 mg/dl were considered as alcohol-related.

The degree of psychomotor impairment resulting from various psychotropic drug concentrations was evaluated by means of a 6-grade scoring system from 0 (no effects) to 6 (probable effects) based on the expected level of impairment for each level of drug reported (see Table 1). For each drowning case, two authors of the present study, both forensic toxicologists (E.V., P.Li.), provided, blinded, a separate score for the effect of each drug, and then a summary score for the overall effects of the drugs. The scoring system was based on the drug concentration with estimated concentration-dependent impairment, taking into account PM changes and redistribution of drugs. The grading was based on that which the National Institute for Health and Welfare in Finland uses when estimating the impairing effects of drugs in traffic [23]. Grades 1 to 3 correspond approximately to impairment observed with BAC under 50 mg/dL. Similarly, grade 4 corresponds to 50 to 119 mg/dL, grade 5 over 120 mg/dL, and grade 6 markedly high BAC values. In case of divergent summary scores (Cohen’s kappa coefficient: 0.77), the two forensic toxicologists held a consensus meeting and approved a consensus score. Only the summary score served for analysis of the results.

**Table 1:** Grading system to evaluate the extent each drug would contribute to drowning

*Grade*	*Effect*
*0*	*None*
*1*	*Not probable*
*2*	*Fairly improbable*
*3*	*Cannot be excluded not confirmed*
*4*	*Cannot be excluded*
*5*	*Quite probable*
*6*	*Probable*

Adapted from Lillsunde P. Blood. In: Drugs and driving - analytical and epidemiological aspects. Publications of the National Public Health Institute; 1996:29–30-31 with author’s permission

## Results

Data extracted from the Helsinki University Toxicological laboratory included 1746 unintentional drownings representing 61.7% of all drownings (unintentional, suicide, homicide, undetermined) examined during the study period at this laboratory. Of these unintentional drownings, 100% were tested for alcohol and 91.5% for other drugs (Table 2). The different percentage of cases tested for alcohol and other drugs occurred because any medical examiner can choose between either full toxicological analysis (alcohol and other drugs) or, alternatively, only alcohol.

**Table 2:** Fatal unintentional drowning, all ages, examined at the Department of Forensic Medicine, University of Helsinki, during the study period (2000–2009)

Drowning	No of cases	Testing rates	
		Alcohol (%)	Medicinal drugs (%)
Unintentional (V90, V92, W65-W74)	1746	100%	91.5%
*Boating-related (V90, V92)*	*303*	*100%*	*91.1%*
*Non-boating (W65-W74)*	*1443*	*100%*	*91.6%*

For all the 1746 unintentional drownings at any age, 1059 (60.7%) tested positive for alcohol and 458 (26.2%) for at least one psychotropic drug. Only the 1697 unintentional drowning victims aged 15 years or older were further considered in this study.

Table 3 summarizes the main toxicological findings in our unintentional drowning victims (see also Additional files 1 and 2).

**Table 3:** Unintentional drowning, 2000–2009: Main toxicological findings in victims aged 15 years or older

Drowning	Overall	BAC ≥ 50 mg/dL		Psychotropic drugs* without alcohol		Psychotropic drugs* and BAC ≥ 50 mg/dL	
	*n*	*n*	%	*n*	%	*n*	%
Unintentional	1697	1058	62.3	134	7.9	313	18.4
*Boating (V90, V92)*	*303*	*197*	*65.0*	*8*	*2.6*	*28*	*9.2*
*Non-boating (W65-W74)*	*1394*	*861*	*61.8*	*126*	*9.0*	*285*	*20.4*

^*^ATC: N01-N06

Boating-related drowning showed the highest percentage to be alcohol-positive (BAC ≥ 50 mg/dL) victims (65.0%). Conversely, psychotropic drugs were more prevalent in victims of non-boating drowning. Almost one-third of these victims had psychotropic drugs in their blood alone or in association with alcohol (BAC ≥ 50 mg/dL). Six cases were psychotropic-drug-positive and had a BAC between 0 and 50 mg/dL, of which one was boating-related and five non-boating-related (Table 4).

**Table 4:** Unintentional drowning, 2000–2009: alcohol-positive (≥ 50 mg/dL) cases, among victims aged 15 years or older, by gender

Drowning	Males			Females			Male to female RR*	
	All	alcohol +		All	alcohol +		All	alcohol +
	*n*	*n*	%		*n*	%		
Unintentional	1487	931	62.6	210	127	60.5	7.1	7.3
*Boating (V90, V92)*	*293*	*190*	*64.8*	*10*	*7*	*70.0*	*29.3*	*27.1*
*Non-boating-related (W65-W74)*	*1194*	*741*	*62.1*	*200*	*120*	*60.0*	*6.0*	*6.2*

^*^rate ratio

The male-to-female rate ratio in alcohol-positive non-boating drowning was 6:1, and in boating-related drowning, the corresponding value was as high as 27:1. Percentages of alcohol-positive cases were, however, similar in both genders.

In psychotropic-drug-positive cases, gender distribution was more even; being approximately 4:1 in non-boating- and 11:1 in boating-related drowning (Table 5). Although the number of drug-positive male drownings was greater than of drug-positive female drownings, the percentage of the latter was substantially higher.

**Table 5:** Unintentional drowning, 2000–2009: psychotropic-drug-positive cases, among victims aged 15 years or older, by gender

Drowning	Males			Females			Male to female RR*	
	All	Drug +		All	Drug +		All	Drug +
	*n*	*n*	%		*n*	%		
Unintentional	1487	362	24.3	210	91	43.3	7.1	4.0
*Boating (V90, V92)*	*293*	*34*	*11.6*	*10*	*3*	*30.0*	*29.3*	*11.3*
*Non-boating-related (W65-W74)*	*1194*	*328*	*27.5*	*200*	*88*	*44.0*	*6.0*	*3.7*

^*^rate ratio

**Fig. 1:**
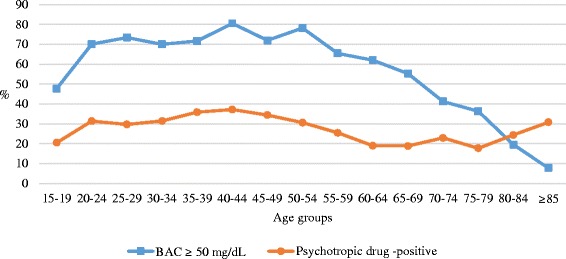
Unintentional drowning, 2000–2009: age-group distribution of percentages of alcohol- and drug-positive cases, victims aged 15 years or older (*n* = 1697*)*

Although no evident age-group peaks occurred for both alcohol- and drug-positive cases, the highest percentage of positive cases for both alcohol- and drug-positive victims was in those 20 to 54 years old. In those over 54, a progressive decrease occurred in the percentage of alcohol-positive victims and, conversely, a fairly stable percentage or even an increase in drug-positive cases among the elderly (Fig. 1).

**Fig. 2:**
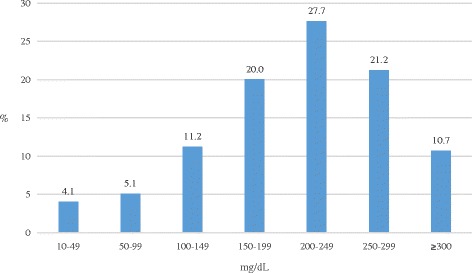
Unintentional drowning, 2000–2009: BAC distribution of percentage of alcohol-positive victims aged 15 years or older (*n* = 1103)

In alcohol-positive victims, the most frequently measured BAC ranged from 200 to 249 mg/dL (305/1103; 27.7%). BAC ranges between 250 to 299 and 150 to 199 mg/dL represented the second and third largest groups, with 234 (21.2%) and 221 (20.0%) victims. BAC was ≥300 mg/dL in 118 victims (10.7%) and below 100 mg/dL in 101 (9.2%) (Fig. 2).

Overall, 70 different psychotropic drugs emerged in 453 unintentional drowning victims aged 15 years or older (Table 6). Of the ten drugs, five (N05BA..) and two (N05C…), and one (N03AF01), are well known for causing psychomotor impairment by mechanisms similar to those of alcohol.

**Table 6:** Unintentional drowning, 2000–2009: the 10 most commonly found psychotropic drugs in victims aged 15 years or older

ATC code/group	Drug	Unintentional drowning victims (n)*
N05BA16/−	desmethyldiazepam**	210
N05BA01/anxiolytic	diazepam	156
N05BA04/anxiolytic	oxazepam	110
N05CD07/anxiolytic	temazepam	99
N06AB04/antidepressant	citalopram	62
N05BA02/anxiolytic	chlordiazepoxide	40
N03AF01/antiepilectic	carbamazepine	30
N05BA12/anxiolytic	alprazolam	18
N05CF01/hypnotic and sedative	zopiclone	18
N06AA09/antidepressant	amitriptyline	16

^*^Some drowning victims were positive for more than one drug, because the number of drugs found was greater than the number of drug-positive cases

^**^metabolite of diazepam and chlordiazepoxide

Almost 20% of unintentional drowning victims presented, along with alcohol association, also with one or more psychotropic drugs. This association was higher in non-boating- than in boating-related drowning (20.4% vs. 9.2%) (Table 3).

### In-depth study: Unintentional drowning, alcohol, and psychotropic drugs

Based on the grading score for psychotropic-drug-positive unintentional drownings in our 453 victims, we distinguished a group 0 (no effects of drugs), as well as drug-affected groups 1 to 3, and 4 to 6 [23].

Among our victims of unintentional drowning, 14.6% tested positive for psychotropic drugs with a grade of 4 to 6 (Table 7). Such high concentrations may actually have contributed to drowning. Cases of this grade were more frequent in non-boating-related drownings, (16.5%) than in boating-related cases (5.6%).

**Table 7:** Unintentional drowning, 2000–2009: psychotropic-drug-positive cases, among victims aged 15 years or older, by grade*

	Overall	Psychotropic-drug-positive*							
		Grade 0		Grade 1–3		Grade 4–6		All grades	
	*n*	*n*	%	*n*	%	*n*	%	*n*	%
All unintentional	1697	2	0.12	204	12.1	247	14.6	453	26.7
*Boating*	*303*	*0*	*0*	*20*	*6.6*	*17*	*5.6*	*37*	*12.2*
*Non boating*	*1394*	*2*	*0.14*	*184*	*13.2*	*230*	*16.5*	*416*	*29.8*

*Grade 0: no effects, Grade 1–3: unlikely role, Grade 4–6: possible to probable role

High drug concentrations (grade 4–6) that may well have contributed to drowning were found in 3.5% (59/1697) of overall unintentional drowning victims who showed no alcohol. Most of these cases were non-boating drownings (Table 8).

**Table 8:** Unintentional drowning, 2000–2009: psychotropic-drug-positive cases with BAC = 0, among victims aged 15 years or older, by grade* (no alcohol)

	Overall	Psychotropic-drug-positive* /no alcohol							
		Grade 0		Grade 1–3		Grade 4–6		All grades	
	*n*	*n*	%	*n*	%	*n*	%	*n*	%
All unintentional	1697	2	0.1	73	4.3	59	3.5	134	7.9
*Boating*	*303*	*0*	*0*	*3*	*1.0*	*5*	*1.7*	*8*	*2.6*
*Non boating*	*1394*	*2*	*0.1*	*70*	*5.0*	*54*	*3.9*	*126*	*9.0*

*Grade 0: no effects, Grade 1–3: unlikely role, Grade 4–6: possible to probable role

Among the 247 unintentional drowning victims graded 4 to 6 for psychotropic drugs, as many as 59 (23.9%) had no alcohol in their blood.

**Fig. 3:**
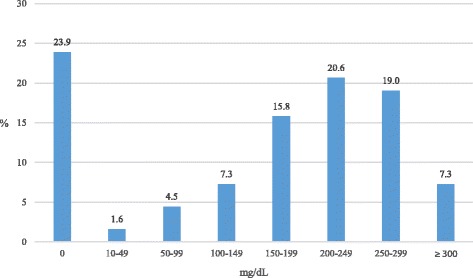
Unintentional drowning, 2000–2009: percentage of victims aged 15 years or older with psychotropic drugs graded 4–6 (Y-axis) (*n* = 247), and their corresponding BAC levels (X-axis)

In 6.1% of the cases graded 4 to 6 for psychotropic drugs (15/247), BAC was positive but <100 mg/dL. The highest association between high psychotropic drug concentration (grade 4–6) and alcohol was in victims having BAC ≥ 100 mg/dL, with a peak for BAC 200 to 249 mg/dL (Fig. 3).

## Discussion

According to WHO standard mortality data and a previous nation-wide survey on drowning, [4] the unintentional drowning rate in Finland is at least three-fold higher than in most Western European and other high-income countries, such as the USA, Australia, and Canada [17]. In Finland, the close association of drowning with alcohol has been addressed repeatedly, [4, 9] and it is the focus of nationwide drowning-prevention campaigns [24]. Statistics Finland (SF) mortality data made available to our research group (permission TK53–277-15), during 2000–2009 include 2001 unintentional drownings with ICD-10 external cause of death codes (V90, V92, W65-W74) (unpublished data). The data set provided by the Laboratory of Forensic Toxicology, Department of Forensic Medicine, University of Helsinki, included 1746 unintentional drownings with ICD-10 external cause of death codes, and therefore includes approximately 87.3% of those reported by SF (see Additional file 3).

The 255 drowning cases for which no toxicological analysis was performed most likely represent a heterogeneous group. This group includes drowning cases for which the medical examiner considered, based for instance on the victim’s age and circumstances, that a role for alcohol and drugs was unlikely. Among the non-tested victims, 123 were under the age of 15 or over 65. Victims dying in hospital may have been under the influence of alcohol and/or drugs during the submersion events. PM toxicology is, however, not usually performed in cases with a prolonged interval between submersion and death in hospital.

Whereas studies on unintentional drowning and alcohol have been performed in high-income countries, only very limited data are available on unintentional drowning and psychotropic drugs, [5, 25] and with the few exceptions of limited case series or single-case reports, [15, 26] no information on drug concentrations has been available.

### Alcohol and drowning

An association between alcohol and unintentional drowning has been highlighted in many studies [4, 8, 27]. In the current study, more than 60% of our non-boating-related drowning victims (ICD-10 W65–74) had a BAC ≥ 50 mg/dL, with the percentage of boating-related drownings (ICD-10 V90, V92) associated with alcohol even higher. Our results are in agreement with other Finnish results [4, 9]. These values, based on data provided by the toxicological laboratory, do not differ markedly from those made available by SF [28] that are based on the mere mention of alcohol as a contributing factor in drowning on the cause-of-death certificate (61.8 vs. 60.8%).

SF does not supply BAC values, however, making evaluation of the causal effects of alcohol on drowning impossible. From the present data, a concentration-effect relationship between alcohol and drowning is apparent. High levels of alcohol thus almost certainly contribute to drowning, while boating, swimming, or engaging in any other activities in aquatic settings. In addition to dose-dependent psychomotor impairment and lowering of the cognitive processes, alcohol may promote risk-taking behaviour. This will likely lead to underestimating and misinterpreting the risks of swimming or boating under challenging circumstances or to neglect of safety regulations: for example, using a life-jacket or –vest [29].

Considering cases with BAC over 50 mg/dL to be alcohol-related reduces the possible bias of PM alcohol production [30]. Future studies should address in depth the actual impact of PM alcohol production in epidemiological studies on drowning [31–33]. The availability in our database of the alcohol concentrations in urine and vitreous humor samples will assist in discriminating PM alcohol production from cases due to alcohol intake. What should, however, be noted is that even a low BAC affects psychomotor and cognitive performance; performance can even be impaired during the hangover period when blood alcohol is no longer detectable in the blood [34, 35].

On the other hand, our data set revealed that as much as 10.7% of the cases had a BAC ≥ 300 mg/dL, a value considered potentially lethal [36, 37]. Although the circumstances are not comparable, it is interesting to note that the peak incidence of alcohol-positive unintentional drowning was found at a BAC between 200 and 249 mg/dL, a situation similar to that reported in research covering land-traffic accidents [38–40]. The high male-to-female ratio for unintentional drowning, especially in alcohol-positive cases, is consistent with the general gender distribution for overall injury deaths, [41] and suggests that males may be more inclined to risk-taking behavior in water-related situations [42]. A thorough analysis of the gender difference among drowning victims should, however, take into account possibly differing gender exposures to risk in water settings [43].

### Drugs and drowning

The correlation of alcohol and drowning emerges in several studies, but the effect of drugs’ contribution to drowning has not been extensively studied. Among the few studies is one in Sweden, during an 18-year study period, in which, of 2075 unintentional- drowning victims, 462 (22%) were positive for psychotropic drugs [5]. A similar study from Ohio, in the USA, found that of 144 accidental drownings, 44 (31%) were negative for alcohol but positive for other drugs, but no separate data were available for psychotropic drugs [25]. In both of these studies, the cases were, however, presented merely as drug-positive or drug-negative, with no concentrations of different types of drugs established.

Here, we focused on psychotropic drugs and their potential involvement in unintentional drowning (1). Concentrations of the medicinal drugs, combined effects of several drugs, and their influence on drowning were evaluated by two expert toxicologists using an ad hoc grading system.

Limitations exist in the grading system and evaluation method we used. Similar grading systems appear in studies conducted in the field of traffic medicine and psychotropic drugs, [44] but the settings and victim’s activities are not comparable. Moreover, the proposed legal levels of drugs for traffic are usually based on scientific evaluation of impairment after a single-dose of a drug administered to each test subject. Furthermore, tolerance phenomena, abnormal metabolism, age, gender, or weight have not been taken into consideration [44].

PM toxicological studies differ from toxicology performed on a living individual, such as in the context of drunken driving or driving under the influence of drugs. In this regard, a relevant aspect is the PM drug redistribution that may challenge the interpretation of PM drug concentration [45, 46]. A better understanding of the actual role of alcohol and psychotropic drugs would result from analysis, in the context of a safety investigation, of all the events underlying and leading to a single drowning event [47]. In addition, consideration of differing circumstances and individual backgrounds, as well as differential effects of fast- and slow-acting psychotropic drugs in water settings would provide further insights.

Interestingly, our level of psychotropic drugs present in one-third of unintentional drowning victims, was a value similar to that reported in Sweden [5]. Psychotropic drugs, as well as alcohol, are present in many individuals in a population, making it important, as regards drowning, not to confuse association with causation. Definitive data on the presence of alcohol or drugs or both in all persons who engage in water-related activities, including those who do not drown, would be helpful. One intrinsic limitation to this study is the absence of such information.

Our findings suggest that psychotropic drugs may play a significant role in drowning, as they did in up to 14.6% of our cases, independently or in association with alcohol. Among our overall unintentional drowning victims, 3.5% were alcohol-negative but had high blood concentrations of one or more psychotropic drugs, making the contribution of psychotropic drugs to such drowning cases possible, even very probable. In four additional cases (0.24%) in which drug concentrations were high and BAC levels were between 10 and 49 mg/dL, the role of psychotropic drugs may well have enhanced the effects of alcohol in the events leading to drowning.

In the remaining 10.8% of our unintentional drowning victims, these had a BAC ≥ 50 mg/dL and a high blood concentration of one or more psychotropic drugs. In these cases, the drugs’ contribution to drowning may also have been possible or even very likely. It is challenging, however, to disentangle the effects of alcohol from those of psychotropic drugs in the events leading to drowning. Psychotropic drugs have known additive effects on the adverse effects of alcohol, [48, 49] making it probable, yet hard to confirm, that without such combined drug and alcohol effects, some of the drownings may have not occurred.

Our findings may have important implications for prevention, when considering the prescribing and use of psychoactive drugs in patients who are keen to engage in swimming and other aquatic activities; especially those likely to use alcohol.

Of our over 70 different psychotropic drugs found, the most common were desmethyldiazepam, diazepam, oxazepam, and temazepam, all of which are benzodiazepines. Impaired cognitive and psychomotor skills are well-known side-effects of benzodiazepines, [48, 50] effects addressed in studies focusing on traffic accidents [13, 51–53]. These adverse effects should be taken into account regarding boating or other recreational or professional activities in aquatic settings.

Whereas psychomotor impairment associated with benzodiazepines can be sufficient to elevate the risk of drowning, among the 10 most common psychoactive and non-psychoactive drugs detected in drowning victims are several known to cause drug-induced long-QT syndrome (DILQTS); this may lead to severe arrhythmias [54–56]. Two antidepressants found in our drowning victims cause relatively less-prominent psychomotor impairment than do benzodiazepines. Citalopram, a member of the serotonin selective reuptake inhibitor (SSRI) class of drugs, and amitriptyline, a tricyclic antidepressant, cause no psychomotor impairment but share the side-effects of prolongation of the QT interval on an electrocardiogram [57]. It should be noted that moderate to high levels of alcohol may also prolong the QT interval, [58, 59] raising the issue of whether mechanisms other than, or in addition to, psychomotor impairment may contribute to such deaths.

Genetically determined long-QT syndromes are associated with sudden cardiac death and drowning, [60, 61] implying that DILQTS, when combined with reflexes activated by actions such as swimming, likewise elevate risk for drowning—as a consequence of fatal arrhythmias [62]. Thus, DILQTS may lead to sudden cardiac death or incapacitation while in water followed by rapid submersion and liquid inhalation. DILQTS may also contribute to life-threatening arrhythmias in the context of the autonomic conflict triggered by parasympathetic and sympathetic activation consequent to rapid immersion in cold water and the diving reflex [62–65]. One recent report documented an association between a high blood level of citalopram and a fatal SCUBA dive likely mediated by DILQTS and the diving reflex [15].

Further steps in investigating the actual role and mechanism of drugs in unintentional drowning will require appropriate consideration of pre-drowning activity, settings, and events leading to death in water. Finally, we will need specific evaluation of whether toxicological findings in victims of unintentional drowning differ from those of the entire population and the subset of the population engaged in water-related activities, as well as the victims of unintentional injury deaths in general, and those of intentional drowning.

## Conclusion

Psychotropic drugs other than alcohol may be, alone or in association with alcohol, an overlooked factor contributing to fatal unintentional drowning in almost 15% of cases. The impact of psychoactive drugs on drowning may be aggravated by the additive effects of alcohol in aquatic settings. The actual mechanism by which psychoactive drugs or alcohol or both combined may contribute to drowning deserves further investigations; the mechanism may have crucial implications for drug prescription and dispensing practices. In addition to impairing cognitive and psychomotor performance in aquatic settings, other mechanisms, such as drug-induced fatal arrhythmias, may contribute to some otherwise unexplained deaths in water.

## Additional files

Additional file 1: Overall drugs found in unintentional drowning, 2000–2009. (DOCX 13 kb)

Additional file 2: Nervous system drugs in unintentional drowning by ATC group and subgroups, 2000–2009. (DOCX 13 kb)

Additional file 3: Fatal unintentional drowning in Finland 2000–2009 by age and gender, according to Statistics Finland and the Laboratory of Forensic Toxicology, University of Helsinki. (DOCX 15 kb)

## Abbreviations

ATC: Anatomical Therapeutic Chemical
BAC: Blood alcohol concentration
CNS: Central nervous system
DILQTS: Drug-induced long-QT syndrome
PM: Post-mortem
SF: Statistics Finland
SSRI: Serotonin selective reuptake inhibitor
WHO: World Health Organization

## Footnotes

1 During the study period illicit drugs were not routinely tested by means of immunological methods. The current practice to perform immunological test for them relied on the victim’s background information and the medical examiner’s suspicion of drug use or need for its exclusion. Similarly to Sweden’s system, fewer than 15 to 20% of all accidental drowning cases were screened for illicit drugs. Based on such screening, we found 10 cases in which blood tested positive for amphetamine and 1 case for methamphetamine. A number of the medicinal drugs detected also have the potential for abuse.
