# Supplementary material for: Unintentional drowning: Role of medicinal drugs and alcohol
Source: BMC Public Health. 2017 May 19;17:388. doi: 10.1186/s12889-017-4306-8 (PMC5437510; doi:10.1186/s12889-017-4306-8)
Supplement: Supplementary file 1 — Overall drugs found in unintentional drowning, 2000–2009. (DOCX 13 kb) [file 12889_2017_4306_MOESM1_ESM.docx]

Additional file 1. Drugs found in unintentional drowning, 2000-2009: Overall, all drugs found by ATC groups

| ATC Group | Name | n* | % |
| --- | --- | --- | --- |
| A | Alimentary tract and metabolism | 22 | 1.6 |
| B | Blood and blood-forming organs | 25 | 1.8 |
| C | Cardiovascular system | 202 | 15.0 |
| J | Anti-infectives for systemic use | 9 | 0.7 |
| M | Musculo-skeletal system | 35 | 2.6 |
| N | Nervous system | 1014 | 75.1 |
| P | Antiparasitic products, insecticides, and repellents | 8 | 0.6 |
| R | Respiratory system | 35 | 2.6 |

ATC= Anatomical Therapeutic Chemical

* Some drowning victims were positive for more than one drug; therefore the number of drugs found is greater than the number of drug-positive cases.
