# Supplementary material for: Unintentional drowning: Role of medicinal drugs and alcohol
Source: BMC Public Health. 2017 May 19;17:388. doi: 10.1186/s12889-017-4306-8 (PMC5437510; doi:10.1186/s12889-017-4306-8)
Supplement: Supplementary file 2 — Nervous system drugs in unintentional drowning by ATC group and subgroups, 2000–2009. (DOCX 13 kb) [file 12889_2017_4306_MOESM2_ESM.docx]

Additional file 2. Unintentional drowning, 2000-2009: the ATC nervous system drugs by sub-group

| ATC Code | Name | n* | % |
| --- | --- | --- | --- |
| N01 | Anaesthetics | 6 | 0.6 |
| N02 | Analgesics | 48 | 4.7 |
| N03 | Antiepileptics | 67 | 6.6 |
| N04 | Anti-parkinson drugs | 3 | 0.3 |
| N05 | Psycholeptics | 729 | 71.9 |
| N06 | Psychoanaleptics | 161 | 15.9 |

* Some drowning victims were positive for more than one drug, making the number of drugs found greater than the number of drug-positive cases.
