# Supplementary material for: Unintentional drowning: Role of medicinal drugs and alcohol
Source: BMC Public Health. 2017 May 19;17:388. doi: 10.1186/s12889-017-4306-8 (PMC5437510; doi:10.1186/s12889-017-4306-8)
Supplement: Supplementary file 3 — Fatal unintentional drowning in Finland 2000–2009 by age and gender, according to Statistics Finland and the Laboratory of Forensic Toxicology, University of Helsinki. (DOCX 15 kb) [file 12889_2017_4306_MOESM3_ESM.docx]

Additional file 3. Fatal unintentional drowning in Finland 2000-2009 by age and gender, according to Statistics Finland and the Laboratory of Forensic Toxicology, University of Helsinki.

| Age group | Statistics Finland | | Laboratory of Toxicology | |
| --- | --- | --- | --- | --- |
|  | Males | Females | Males | Females |
| > 15 | 55 | 23 | 29 | 20 |
| 15-19 | 45 | 2 | 41 | 3 |
| 20-24 | 55 | 6 | 58 | 9 |
| 25-29 | 64 | 3 | 61 | 3 |
| 30-34 | 66 | 5 | 66 | 4 |
| 35-39 | 99 | 13 | 94 | 12 |
| 40-44 | 114 | 16 | 98 | 15 |
| 45-49 | 160 | 25 | 139 | 18 |
| 50-54 | 191 | 29 | 171 | 22 |
| 55-59 | 224 | 31 | 192 | 28 |
| 60-64 | 182 | 26 | 171 | 24 |
| 64-69 | 167 | 33 | 140 | 30 |
| 70-74 | 144 | 21 | 119 | 12 |
| 75-79 | 113 | 24 | 91 | 22 |
| 80-84 | 46 | 4 | 38 | 3 |
| ≥85 | 10 | 5 | 8 | 5 |

Note: Few cases included in the database of the Laboratory of Forensic Toxicology may not be included in the Statistics Finland database. Foreign non-resident victim of drowning generally undergo medico-legal autopsy (and post-mortem toxicology) but are not included in the Statistics Finland database.
